# Nuclear group I introns in self-splicing and beyond

**DOI:** 10.1186/1759-8753-4-17

**Published:** 2013-06-05

**Authors:** Annica Hedberg, Steinar D Johansen

**Affiliations:** 1 RNA lab-RAMP, Department of Medical Biology, Faculty of Health Sciences, University of Tromsø, Tromsø N-9037, Norway

**Keywords:** Catalytic introns, *Diderma*, *Didymium*, Group I introns, Intron biology, Intron mobility, *Physarum*, RNA processing, *Tetrahymena*

## Abstract

Group I introns are a distinct class of RNA self-splicing introns with an ancient origin. All known group I introns present in eukaryote nuclei interrupt functional ribosomal RNA genes located in ribosomal DNA loci. The discovery of the *Tetrahymena* intron more than 30 years ago has been essential to our understanding of group I intron catalysis, higher-order RNA structure, and RNA folding, but other intron models have provided information about the biological role. Nuclear group I introns appear widespread among eukaryotic microorganisms, and the plasmodial slime molds (myxomycetes) contain an abundance of self-splicing introns. Here, we summarize the main conclusions from previous work on the *Tetrahymena* intron on RNA self-splicing catalysis as well as more recent work on myxomycete intron biology. Group I introns in myxomycetes that represent different evolutionary stages, biological roles, and functional settings are discussed.

## Review

### Introduction

Introns are genetic elements that interrupt functional RNA- or protein-coding genes, and are removed post-transcriptionally in a process termed splicing. Their ability to be spliced out at RNA level makes them almost invisible for the host and limits the phenotypic cost, and introns have often been labeled selfish elements or molecular parasites [1]. A major class of introns is represented by the self-splicing group I introns. These introns are widespread but sporadically distributed in nature, and they are present in the genomes of some bacteria, mitochondria, chloroplasts, bacteriophages, and eukaryotic viruses, and in the nuclei of eukaryotic microorganisms [2].

Group I introns in nuclear genomes are exclusively found within functional ribosomal RNA (rRNA) genes of a wide spectrum of eukaryotic microorganisms. Here they are frequently noted among red algae, chlorophyte algae, fungi, and myxomycetes, but only occasionally in ciliates [2-4]. Nuclear group I introns interrupt universally conserved sequences in the small subunit (SSU) and large subunit (LSU) rRNA genes. About 100 ribosomal DNA (rDNA) insertion sites have been noted to contain introns, 50 in the SSU and 50 in the LSU rRNA genes [3,4]. Interestingly, each insertion site appears to harbor at least one distinct family of group I introns with a separate evolutionary history [2,5,6], which probably reflects site-specific intron mobility (see below).

Despite the fact that thousands of nuclear group I introns have been annotated in sequence databases, only a very few have been submitted to molecular analyses and functional characterizations. The *Tetrahymena* LSU rRNA intron at insertion site L1925 has become the undisputedly most important group I intron model system for the study of RNA catalysis, RNA structure, and RNA folding [7]. Tth.L1925 (see [8] for nuclear group I intron and rDNA insertion site nomenclature) was discovered and reported more than 30 years ago within the extrachromosomal rDNA of the ciliate *Tetrahymena thermophila *[9]. This *Tetrahymena* intron has been thoroughly investigated and self-splicing was shown to occur by RNA catalysis based on a two-step transesterification reaction requiring a guanosine cofactor [7].

**Figure 1:**
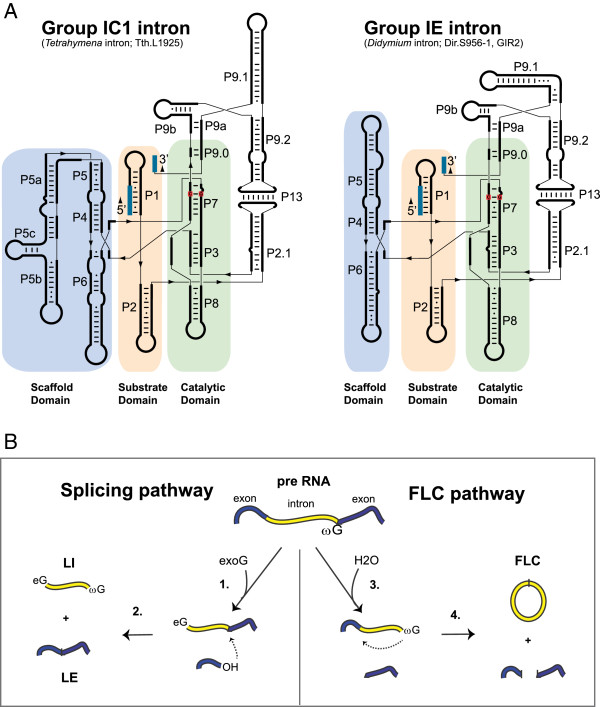
**Secondary structures and processing pathways of nuclear group I intron RNAs.** (**A**) Secondary structure diagrams of the group IC1 intron ribozyme in *Tetrahymena* (Tth.L1925) and the group IE intron ribozyme in *Didymium* (Dir.S956-1, GIR2). The paired segments (P1 to P9, P13) are noted. The three core domains and the conserved G-C pair at P7 are highlighted. The 5’ and 3’ exons are shown in blue. (**B**) The two main processing pathways, self-splicing and full-length intron circularization (FLC). The self-splicing pathway involves two transesterification reactions. The first reaction is initiated by a nucleophilic attack by the hydroxyl group of an exogenous guanosine cofactor (exoG) (1). The second transesterification reaction starts with a nucleophilic attack at the 3’ splice site (SS) (2), resulting in ligated exons (LEs) and linear intron (LI) RNA molecules. The FLC pathway involves hydrolysis and transesterification reactions. A hydrolytic cleavage at the 3’ SS (3) is followed by a nucleophilic attack at the 5’ SS by the terminal guanosine (ωG) (4) resulting in a full-length intron circle and non-ligated exons. The FLC pathway is independent of exoG. eG: exogenous guanosine factor covalently linked at the 5’ end of the free intron RNA; exoG: exogenous guanosine cofactor; FLC: full-length circularization; LE: ligated exon; LI; linear intron; SS: splice site.

Further structural investigations have revealed a well-defined and highly conserved RNA core responsible for the catalysis, despite the fact that almost no universally conserved nucleotide residues are present among group I introns. Nuclear group I introns are mainly represented by two of the five subgroups, the group IC1 and group IE [6,10], and schematic drawings of secondary structures are shown in Figure 1A. The functional RNA part of the intron, the group I ribozyme core, consists of about nine paired segments (named P1 to P9), as well as one or more optional segments (for example, P10 and P13). These helices are further organized into three helical stacks referred to as the catalytic domain (P3 and P7, proximal P8 and P9), the substrate domain (P1 and proximal P2), and the scaffold domain (P4, P5 and P6) [11]. The domains are easily recognized in both the group IC1 and group IE introns, but with some notable differences. The group IC1 ribozyme, represented by the *Tetrahymena* intron, has a more complex structured scaffold domain than the *Didymium* group IE ribozyme (Figure 1A). Crystal structure analysis of the *Tetrahymena* ribozyme core has revealed a highly compact RNA architecture where the substrate domain is docked into a narrow cleft made by the catalytic domain wrapping around the scaffold domain [7,11,12]. The substrate specificity is in part dependent on a 4 to 6 nucleotide base pairing (P1) between the internal guide sequence and the 5′ exon, and the guanosine binding site (G site) is located in the P7 helix where a conserved G-C pair is the main component (Figure 1A).

A small fraction of the nuclear group I introns have the potential of being mobile elements since they harbor large homing endonuclease gene (HEG) insertions. The HEGs are located within the peripheral parts of the paired segments such as P1, P2, P6, P8 or P9, and expression of these protein-coding genes embedded in nucleolar rDNA utilizes unconventional strategies [13]. Interestingly, both sense and antisense HEG organizations relative to the group I ribozymes have been reported [6,14,15].

Several group I intron-based biotechnological applications have been suggested and realized. These involve the intron homing endonuclease (HE) as a highly specific and rare-cutting endonuclease, intron splicing as a therapeutic tool in fighting pathogens and engineered group I ribozymes as molecular tools in RNA reprogramming and RNA repair [16,17]. Ribozymes have features that can be modified and used in several applications involving gene regulation analysis and gene therapy [17,18]. These approaches are based on engineered *trans*-splice group I ribozymes, and most studies have been performed with the *Tetrahymena* ribozyme. One of the best characterized examples of RNA-based gene repair is the reprogramming of mutant p53 transcripts in human cancer cells [19,20]. The reprogramming includes an engineered ribozyme, which replaces a defective RNA sequence with a functional p53 homolog [21]. However, the low specificity and low efficiency are important limitations in further development of group I ribozymes in biotechnology [17].

**Figure 2:**
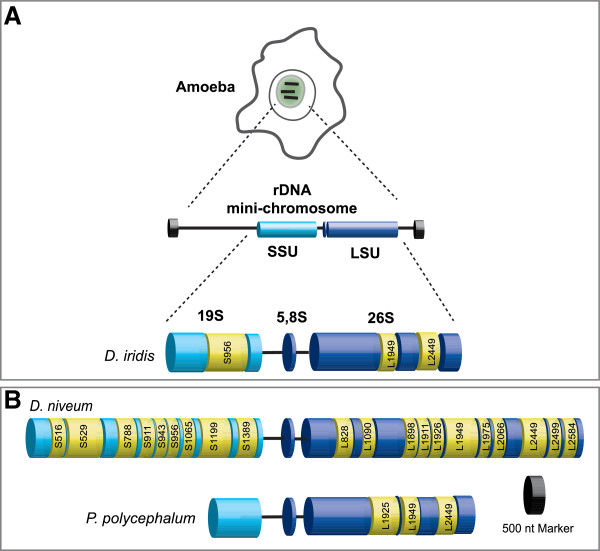
**Group I introns in extrachromosomal nuclear rDNA of myxomycetes.** (**A**) The rDNA mini-chromosomes are located within the nucleolus of myxomycetes. The multicopy mini-chromosomes of *Didymium iridis* contain the SSU and LSU rRNA genes and have regular telomeres at the ends. The rRNA genes harbor three group I introns (yellow). The S956 intron in the SSU is self-splicing and contains HEG, while the two introns (L1949 and L2449) in the LSU are obligatory introns dependent on the host for splicing. In fact, two versions of *D. iridis* S956 have been described: the twin-ribozyme intron S956-1 in the Panama 2 isolate and S956-2 in the Costa Rica 8 isolate with antisense HEG orientation. (**B**) The location of the group I introns within the SSU and LSU are shown for the *Diderma niveum* Italian isolate and the *Physarum polycephalum* Carolina isolate. All species contain the obligatory introns L1949 and L2449. The 20 group I introns found in *D. niveum* have four main categories. *P. polycephalum* also contains a mobile intron at position L1925 encoding I-*Ppo*I. For nomenclature of rDNA introns and insertion sites, see [8]. HEG: homing endonuclease gene; LSU rRNA: large subunit ribosomal RNA; nt: nucleotide; rDNA: ribosomal DNA; SSU rRNA: small subunit ribosomal RNA.

Whereas the *Tetrahymena* intron has proven to be the undisputed prototype in the study of RNA catalysis and RNA structure, other nuclear group I introns have contributed to our understanding of intron functions beyond splicing. Recent studies have shown that the extrachromosomal nuclear rDNA of myxomycetes, eukaryotic microorganisms belonging to the Amoebozoa clade, contain an abundance of group I introns (Figure 2) [4,5,22]. Currently about 500 nuclear group I introns in myxomycetes have been described, and these introns are amazingly diverse in sequence, structure, organization and insertion sites. About 10% of the myxomycete rDNA introns harbor HEGs, and most group I introns tested are able to self-splice as naked RNA *in vitro* without any essential assistance from host factors.

Three myxomycete species have been investigated in detail (Figure 2). *Physarum polycephalum* contains the intron (Ppo.L1925), which is cognate to that of the *Tetrahymena* intron; it harbors a HEG and is mobile in genetic crosses between intron-lacking and intron-containing strains [23,24]. The most complex organized nuclear group I intron known is the twin-ribozyme intron (Dir.S956-1) in *Didymium iridis*. Dir.S956-1 is mobile in genetic crosses and contains two distinct ribozymes with different functions in splicing and RNA processing, as well as a HEG [25,26]. A second variant of the *Didymium* intron (Dir.S956-2) harbors a HEG at the antisense orientation [15,27]. Finally, *Diderma niveum* has an extremely dense intron content with 20 or more group I introns present within the same rRNA primary transcript (Figure 2B) [22,28]. The myxomycetes *P. polycephalum*, *D. iridis* and *D. niveum* have all undergone whole genome sequencing analysis, including their rDNA mini-chromosomes and corresponding introns ([4,29]; our unpublished results).

Here we summarize the major hallmarks of nuclear group I intron catalysis and mobility based on key model introns in *Tetrahymena*, *Physarum*, and *Didymium* rDNAs. We then discuss the functional implications of different categories of introns and provide representative examples from *Diderma*. Finally, we present an example of a group I intron that recently has gained a new molecular function and biological role.

### Group I ribozyme reactions

Group I intron RNAs catalyze transesterification and hydrolysis reactions, and the detailed mechanisms have been extensively reviewed [7,28,30]. These reactions involve two main processing pathways, splicing and full-length intron circularization (Figure 1B), which are parallel and mutually exclusive, and result in different end products [28]. The *Tetrahymena* intron has been the prototypical ribozyme in the study of the splicing pathway. Self-splicing depends on two consecutive transesterification reactions initiated by a nucleophilic attack of the 3′OH of an exogenous guanosine cofactor (exoG) at the 5′ splice site (SS) (Figure 1B). ExoG is specifically bound to the P7 catalytic core segment of the splicing ribozyme prior to the first splicing step. This reaction leaves exoG covalently attached to the 5′ end of the intron RNA as well as a free 5′ exon with an available 3′OH group. In the second transesterification reaction, exoG is replaced by the terminal guanosine (ωG) at P7, and the reaction is initiated when the 5′ exon attacks the 3′ SS, resulting in ligated exons and the released linear intron. *In vitro* studies of the *Tetrahymena* intron have shown that the linear intron RNA may undergo additional circularization reactions leaving a variety of truncated circles [31,32]. However, the biological significance of truncated intron circles, if any, is unclear.

The full-length intron circularization (FLC) pathway has been studied in detail for the *Didymium* group I intron Dir.S956-1 [33]. This pathway is common among self-splicing nuclear group I introns and is initiated by a hydrolytic cleavage at the 3′ SS [34]. The 3′OH of the ωG then attacks the 5′ SS resulting in a covalently linked full-length circular intron (Figure 1B). Interestingly, the FLC pathway produces fragmented RNA exons, which are unligated and expected to produce non-functional rRNAs.

### Group I intron mobility at the DNA or RNA level

**Figure 3:**
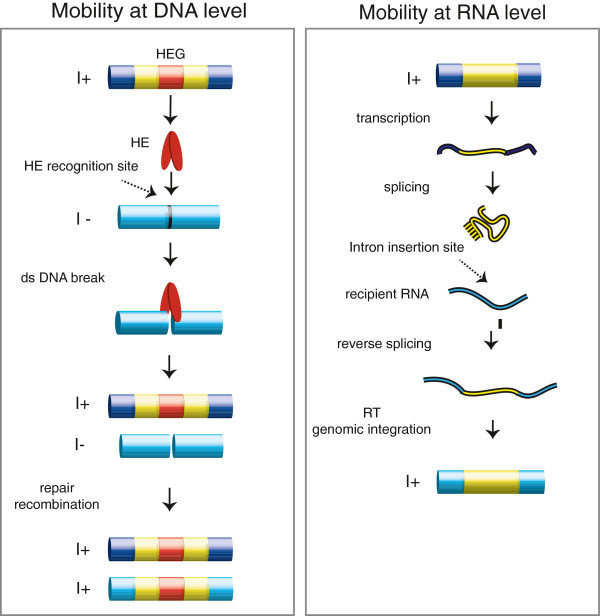
**Group I intron mobility.** Mobility at the DNA level (left) involves a dsDNA break by the homing endonuclease (HE) at the HE recognition site followed by recombination and repair. HE-dependent mobility is unidirectional and highly efficient. Mobility at the RNA level (right) involves reverse splicing into an intron insertion site in recipient RNA molecules followed by reverse transcription and genomic integration (see text for details). Exon sequences are indicated by blue cylinders (DNA) and lines (RNA), and introns by yellow cylinders (DNA) and lines (RNA). HE: homing endonuclease; HEG: homing endonuclease gene; I+: intron-containing allele; I–: intron-lacking allele; RT: reverse transcriptase; dsDNA: double stranded DNA.

The spreading of group I introns may occur at either the DNA or RNA level (Figure 3). The most efficient mobility process is homing at the DNA level, which is initiated by a double-strand break performed by the intron-encoded HE close to, or at, the site of intron insertion at an intron-lacking allele [35]. HE-mediated group I intron homing involves a homology-dependent gene conversion event and results in the unidirectional spread of group I introns at the population level (Figure 3). Only a few nuclear HEs have been further characterized, and these include I-*Ppo*I from *P. polycephalum*[36], I-*Dir*I and I-*Dir*II from *D. iridis *[27], and some isoschizomeric HEs from related *Naegleria* species [37]. Nuclear HEs all belong to the His-Cys family [38,39] and require specific recognition sequences of 15 to 20 bp spanning the intron insertion site.

Experimental evidence for homing in a biological setting has been collected from only two nuclear group I introns, both in the myxomycetes. The first system to be characterized was Ppo.L1925 in *P. polycephalum*, which encodes I-*Ppo*I [23]. Here mobility was shown in mating experiments between intron-containing and intron-lacking amoeba cells. Similarly, homing was also detected in *D. iridis* for the Dir.S956-1 intron [40]. In addition, homing of nuclear group I introns has also been detected in yeast in artificial experimental settings using I-*Ppo*I and the introns Ppo.L1925 and Tth.L1925 (*Tetrahymena* intron), which were integrated into all the approximately 150 genomic rDNA copies at chromosome XII in an elegant experiment [41-43].

It has been suggested that intron homing also occurs directly at the RNA level by reverse splicing. Here, an excised intron attacks the ligated exons at the intron-lacking cognate insertion site and integrates into the precursor RNA. Reverse splicing has been reported *in vitro*, in yeast and in *Escherichia coli* for both the *Tetrahymena* intron [44-46] and the *Didymium* intron [47]. Interestingly, *in vitro* integration of full-length circular intron RNA has also been noted, suggesting a biological role for the circularization pathway in propagation and intron spread [47]. Less frequently, reverse splicing may lead to intron spread at novel rRNA sites, and may explain the low frequency transposition features of nuclear group I introns observed in phylogenetic studies [5,22,48]. However, experimental evidence of the complete pathway including reverse transcription and genomic integration into rDNA is still lacking.

### From parasitism to mutualism: lessons learned from the myxomycete group I introns

Recent studies of the rDNA mini-chromosome in myxomycetes have revealed more than 500 group I introns highly divergent in sequence, size and insertion site. Myxomycetes are eukaryotic microorganisms with a complex life cycle, which has several stages from haploid amoebae cells to a multi-nucleated plasmodium with synchronously dividing diploid nuclei [49]. The rDNA loci are exclusively located on extrachromosomal non-Mendelian mini-chromosomes (Figure 2A). For the myxomycete introns, we summarize results from *P. polycephalum*, *D. iridis* and *D. niveum* rDNAs.

The rDNA from *D. niveum* is highly unusual since at least 20 group I introns are present (Figure 2B). Recent deep-sequencing experiments of paired-end DNA libraries, performed on the SOLiD platform, have confirmed that all introns are present in all rDNA copies of *D. niveum* (our unpublished results). The high abundance of introns is a significant challenge to the host cell since the majority of group I introns have the ability to perform FLC and generate fragmented exons [22]. The myxomycete group I introns can be divided into four main categories based on splicing, mobility and pattern of occurrence.

#### Self-splicing HEG-containing introns

**Figure 4:**
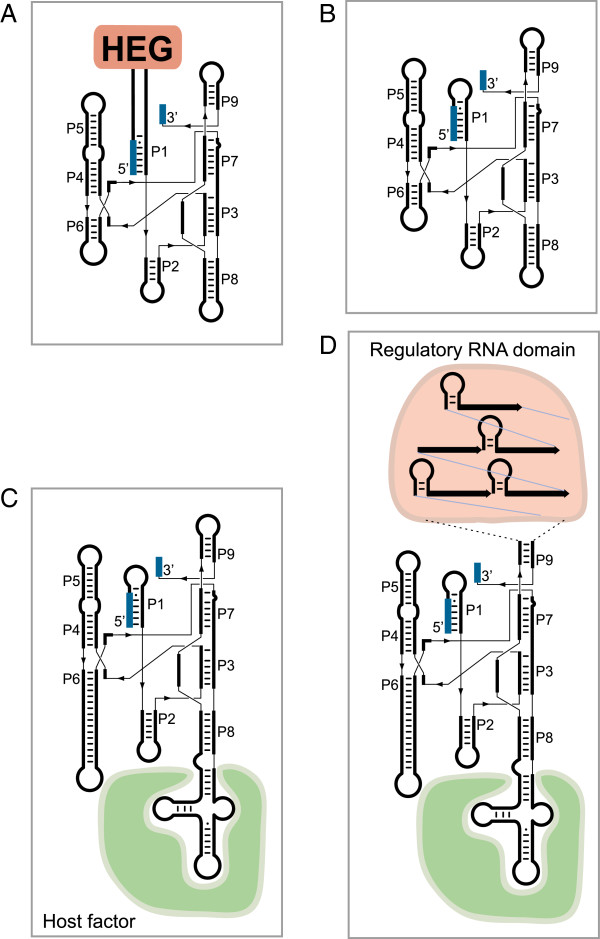
**Schematic structural organization of the four main categories of group I introns observed in myxomycete rDNA.** (**A**) Self-splicing and mobile introns with homing endonuclease genes (HEGs). (**B**) Self-splicing all-ribozyme introns without HEG. (**C**) Optional host-dependent introns with structural extensions available for host factors (green), here exemplified as associated with P8. (**D**) Obligatory host-dependent introns with large extension either associated with host factors and/or containing direct repeats (P9) or other motifs with the potential for being regulatory RNA domains (pink). HEG: homing endonuclease gene; rDNA: ribosomal DNA.

The first category consists of the mobile HEG-containing introns (Figure 4A). Of the representative introns presented in Figure 2, five belong to this category (two different S956 introns in *Didymium*, L1925 in *Physarum*, and S529 and S1199 in *Diderma*). HEGs have been noted in both sense and antisense orientations compared to the group I ribozyme and have been inserted at various locations (P1, P2, P6, P8 and P9). The HEG-containing introns belong to both the IC1 and IE subclass, are highly mobile in biological settings [23,40], and optional among strains and isolates of the same species [27]. Introns in this category are selfish parasitic introns.

#### Self-splicing all-ribozyme introns

Most introns in *Diderma* belong to this category (as well as the *Tetrahymena* intron), and it has been suggested that the all-ribozyme group IC1 and IE introns are remnants of mobile introns after loss of HEGs (Figure 4B). This scenario is founded on the Goddard–Burt cyclic model based on intron invasion, degeneration and subsequent loss [50], which is well supported by several independent reports [51-53]. All-ribozyme introns are usually optional among strains and closely related species. Two of the *Diderma* introns deviate from the universal consensus features of group I introns, but still self-splice as naked RNA *in vitro*. S529 is inserted after a G-residue in rDNA (the U-residue is the consensus) and thus the U:G pair at the 5′ SS is replaced by a G:C pair [54]. Furthermore, the ωG in L2066 is replaced by ωA but still performs efficient and complete self-splicing *in vitro*[22].

#### Host-factor-dependent optional introns

Group I introns sometimes escape the Goddart–Burt cycle [28] and become dependent on host factors for splicing (Figure 4C). An example of an optional host-dependent intron in *Diderma* rDNA is S1389 (Figure 2B). This intron is commonly found among the myxomycete family Didymiaceae, but differs from most myxomycete group I introns as it does not self-splice as naked RNA *in vitro *[55]. One typical feature for the host-dependent splicing introns is extended peripheral loop regions (Figure 4C). In the case of S1389 there are significant size variations in the substrate domain (P1, P2) and the catalytic domain (P8, P9), but the scaffold domain is surprisingly uniform [55]. These structural and functional hallmarks resemble that of mitochondrial fungal group I introns dependent on splicing maturases [56-58]. However, unlike the fungal introns, no specific maturase has yet been identified or characterized for a nuclear group I intron.

#### Host-factor-dependent obligatory introns

Stable long-term relationships between a group I intron and its host have been noted in plant chloroplast genomes (trnL-intron) [59] and in hexacoral mitochondrial genomes (ND5-717 intron) [53]. A third example is found among nuclear group I introns in Physarales myxomycetes. L1949 and L2449 are present in LSU rDNA of all 60 species and isolates investigated of the Physarales families Didymiaceae and Physaraceae; they have been shown to be strictly vertically inherited [4,5,60-62]. None of the L1949 and L2449 introns tested self-splice *in vitro* as naked RNA and thus appear to be dependent on host factors for splicing. Further support for this notion is that a large subset of L1949 introns possesses a truncated catalytic core lacking the important P8 segment [5,60,61]. Another unusual feature of L1949 and L2449 introns is large sequence insertions at peripheral loop regions. No detectable protein-coding capacity can be found, but these large insertions sometimes contain complex direct-repeat motifs [4,62] (Figure 4D). The peripheral regions can, by duplications, increase in size over time and result in large introns [4,62].

Obligatory introns could have gained new mutual functions that benefit the host, and one possibility is that peripheral insertions are further processed into long non-coding RNAs (lncRNAs). lncRNAs are known to regulate gene expression, translation, splicing and trafficking by acting as guides, scaffolds, decoys or enhancers [63,64], and are present in all eukaryote systems investigated. Interestingly, recent 454 pyrosequencing analysis on the *D. iridis* transcriptome revealed stable and differentially expressed L2449 intron RNA in four different life stages of myxomycetes (our unpublished results), which opens the possibility that intron RNA may have gained additional functions beyond splicing.

### Group I intron ribozyme that evolved a new biological role

**Figure 5:**
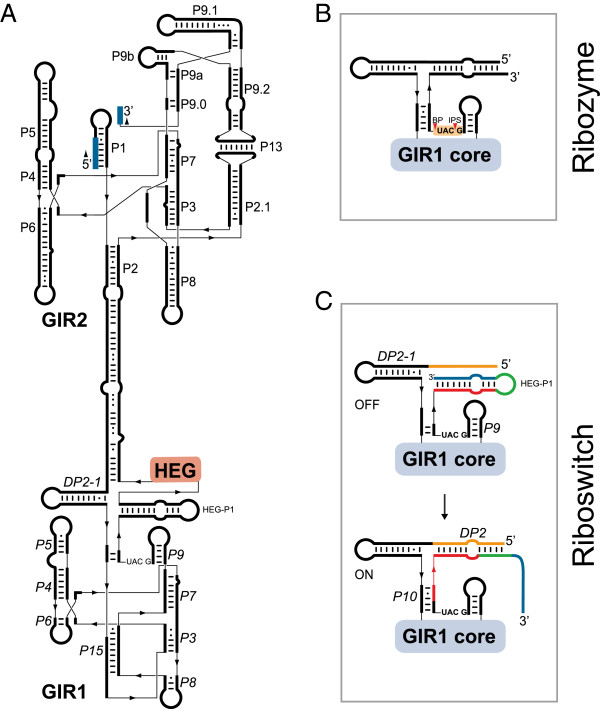
**Structure diagrams of different stages of the GIR1 lariat capping ribozyme derived from a complex twin-ribozyme group I intron.** (**A**) Twin-ribozyme intron (Dir.S956-1) from the *D. iridis* Panama 2 isolate. A standard group IE splicing ribozyme (GIR2; Figure 1B) contains an insertion in helix P2, which consists of a homing endonuclease gene (HEG) and the lariat capping group I-like ribozyme (GIR1). (**B**) The active GIR1 conformation performs a transesterification reaction at the junction between *P9* and *P10* resulting in a 3-nucleotide 2’,5’ lariat structure at the 5’ end of the HE messenger. (**C**) The regulatory domain of GIR1 resembles a complex riboswitch, which alternates between a catalytic inactive GIR1 (containing HEG-P1; the off state) and an active GIR1 conformation (containing *DP2* and *P10*; the on state). This rearrangement involves replacement of RNA structures (color coded). BP: branch point; GIR1: group I-like ribozyme; GIR2: group I splicing ribozyme; HE: homing endonuclease; HEG: homing endonuclease gene; IPS: internal processing site.

One notable example of group I introns that have evolved new biological roles are the twin-ribozyme introns, which are interrupting SSU rRNAs in the myxomycete *Didymium*, the amoebo-flagellates *Naegleria* and the amoeba *Allovahlkampfia *[26,60,65,66]. Twin-ribozyme introns have a highly complex structural organization that consists of a standard self-splicing ribozyme responsible for SSU rRNA exon ligation, intron excision and the generation of full-length intron RNA circles. Furthermore, the splicing ribozyme contains a large insertion in one of its peripheral helices. A schematic structural diagram of the best studied twin-ribozyme intron, Dir.S956-1 in *D. iridis*, is presented in Figure 5A. This *Didymium* intron carries a HEG inserted at P2 of the splicing ribozyme, and is mobile at the DNA level in genetic crosses between intron-containing and intron-lacking strains [26,40].

Interestingly, a second ribozyme domain is located immediately upstream of the HEG. Detailed structural analysis has shown that the ribozyme is derived from a standard group I intron; it is named GIR1 (group I-like ribozyme 1) [67]. GIR1 lacks the 5′ and 3′ SSs, and has unique structural rearrangements in the catalytic core. GIR1 has an essential role in the expression of the intron HEG, which is transcribed by RNA polymerase I and embedded in the rDNA [14]. Recent reports have suggested dual functions for GIR1 in HEG expression: as a capping ribozyme [68] and as a riboswitch regulator [69,70].

#### Lariat capping ribozyme

The catalytic part of GIR1 has about 180 to 200 nucleotides, and when activated it catalyzes self-cleaving by branching [71]. This reaction is highly unusual for the group I ribozymes, but similar to that of group II ribozymes and the spliceosome [68]. GIR1 generates a 3-nucleotide lariat cap by joining the C residue at the internal processing site (IPS) and the U residue at the branch point (BP) by a 2′,5′ phosphodiester bond (Figure 5B) [68]. Similar lariat caps are generated by the *Naegleria* and *Allovahlkampfia* GIR1s [66,72].

#### On-off riboswitch

During the transcription and self-splicing of a twin-ribozyme intron, GIR1 has to be in an inactive conformation to avoid premature cleavage of the rRNA precursor [69]. Thus, the regulatory domain of GIR1 folds into an inactive off-state confirmation, which involves the HEG-P1 helix 3' of the BP (Figure 5C). The excised intron RNA then activates GIR1 into an on state by a conformational change in the regulatory domain [69]. HEG-P1 is replaced by two additional helical segments, *DP2* and *P10*, which depend on base pairing of sequences 5′ and 3′ of the GIR1 core (Figure 5C). Related conformational changes, but differently organized, occur in the *Naegleria* and *Allovahlkampfia* GIR1s [66]. The on-off switch of GIR1 resembles that of many riboswitches [73], but currently no specific ligand has been identified.

## Conclusions

Self-splicing, RNA structure and folding, and HE-dependent homing are fully described features of the group I introns in *Tetrahymena* and *Physarum* rDNA, but these studies represent only part of the story for nuclear group I introns. Additional studies have shown that there exist two main catalytic pathways for intron RNA: the intron splicing pathway and the intron FLC pathway. Intron homing is also represented by two distinct mechanisms: HE-dependent homing and the less efficient reverse-splicing-dependent homing. The latter mechanism sometimes results in intron insertion at non-allelic sites. The next important challenge is to understand the biological role of nuclear group I introns, and a first step has been achieved for the myxomycete protists, which appear to contain an abundance of diverse catalytic rDNA introns. Four main intron categories have been identified, from the true selfish HEG-containing and mobile group I introns, to introns that have become biochemically dependent on the host cell for splicing. Some introns appear obligatory for the host, and intron RNAs may evolve further to gain more regulatory functions. Finally, the lariat capping ribozyme (GIR1) is a unique example of a group I intron that has gained new catalytic properties and new biological roles in nuclear gene regulation.

## Abbreviations

BP: branch point; dsDNA: double stranded DNA; eG: exogenous guanosine factor covalently linked at the 5’ end of the free intron RNA; exoG: exogenous guanosine cofactor; FLC: full-length circularization; GIR1: group I-like ribozyme; GIR2: group I splicing ribozyme; HE: homing endonuclease; HEG: homing endonuclease gene; I+: intron-containing allele; I–: intron-lacking allele; IPS: internal processing site; LE: ligated exon; LI: linear intron; LncRNA: long non-coding RNA; LSU rRNA: large subunit ribosomal RNA; Nt: nucleotide; RDNA: ribosomal DNA; RT: reverse transcriptase; SOLiD: sequencing by oligo ligation and detection; SS: splice site; SSU rRNA: small subunit ribosomal RNA.

## Competing interests

The authors declare that they have no competing interests.

## Authors’ contributions

AH and SDJ contributed equally to the writing for this article. Both authors read and approved the final manuscript.
